# Re-recognizing micro locations of nanoscale zero-valent iron in biochar using C-TEM technique

**DOI:** 10.1038/s41598-021-84685-w

**Published:** 2021-03-03

**Authors:** Kun Yang, Jialu Xu, Ming Zhang, Daohui Lin

**Affiliations:** 1 Department of Environmental Science, Zhejiang University, Hangzhou, 310058 China; 2 Key Laboratory of Environmental Pollution and Ecological Health of Ministry of Education, Hangzhou, 310058 China; 3 Zhejiang Provincial Key Laboratory of Organic Pollution Process and Control, Hangzhou, 310058 China; 4 Department of Environmental Engineering, China Jiliang University, Hangzhou, 310028 China

**Keywords:** Environmental impact, Environmental sciences, Environmental chemistry, Pollution remediation

## Abstract

Biochar supported nanoscale zero-valent iron (NZVI/BC), prepared commonly by liquid reduction using sodium borohydride (NaBH_4_), exhibits better reduction performance for contaminants than bare NZVI. The better reducing ability was attributed to attachment of nanoscale zero-valent iron (NZVI) on biochar (BC) surface or into the interior pores of BC particles due to observations by scanning electron microscopy (SEM) and plan transmission electron microscopy (P-TEM) techniques in previous studies. In this study, cross-sectional TEM (C-TEM) technique was employed firstly to explore location of NZVI in NZVI/BC. It was observed that NZVI is isolated from BC particles, but not located on the surface or in the interior pores of BC particles. This observation was also supported by negligible adsorption and precipitation of Fe^2+^/Fe^3+^ and iron hydroxides on BC surface or into interior pores of BC particles respectively. Precipitation of Fe^2+^ and Fe^3+^, rather than adsorption, is responsible for the removal of Fe^2+^ and Fe^3+^ by BC. Moreover, precipitates of iron hydroxides cannot be reduced to NZVI by NaBH_4_. In addition to SEM or P-TEM, therefore, C-TEM is a potential technique to characterize the interior morphology of NZVI/BC for better understanding the improved reduction performance of contaminants by NZVI/BC than bare NZVI.

## Introduction

Nanoscale zero-valent iron (NZVI), as an environmental friendly material has been widely used in recent decades to reduce contaminants such as Cr(VI), As(V), nitrates, and chlorinated organics^1–6^. Biochar (BC) supported NZVI (NZVI/BC), prepared commonly by liquid-phase reduction method using sodium borohydride (NaBH_4_) as reducing agent, has better ability in reducing pollutants than NZVI^7–9^. The oxidation and agglomeration of NZVI can be effectively suppressed by BC, therefore, NZVI’s surface energy and reduction activity can be maintained^5,10–12^. In previous studies^13–16^, by scanning electron microscopy (SEM) and plan transmission electron microscopy (P-TEM) techniques, it was observed that NZVI particles were loaded on the surface or in the interior pores of BC particles, which is responsible for the improved reduction ability of NZVI^17–19^. Several possible ways were suggested to interpret the loading of NZVI on BC surface or in BC interior pores (i) Fe^2+^/Fe^3+^ ions are adsorbed on surface or into interior pores of BC and then be reduced to NZVI by NaBH_4_^15,20–22^; (ii) Fe^2+^/Fe^3+^ ions are hydrolyzed into precipitates of iron hydroxide, and then deposited on surface or into interior pores of BC particles for reduction to NZVI by NaBH_4_^23^; (iii) Fe^2+^/Fe^3+^ ions are reduced to NZVI by NaBH_4_ in solutions and then deposited on BC surface or into the interior pores of BC particles^20,21^.

SEM and P-TEM are widely used electron microscopy techniques with high resolution and high magnification to visualize the morphology of NZVI/BC as well as the location of NZVI^24–26^. However, by collecting secondary electrons reflected from sample surface, SEM technique can only visualize the sample top surface but not the interior structures^27,28^. For P-TEM technique, the accumulated signals of the sample from top to bottom including the interior structures can be obtained by collecting electrons through the sample^29^. However, the accumulated signals of P-TEM are difficult to explore the interior structure of samples clearly because the interior structure signals are actually covered by the electron signals of top and bottom surfaces, i.e., there is a problem of superimposition for P-TEM technique^30,31^. Both of SEM and P-TEM technique cannot explore the interior structure signals of NZVI/BC particle clearly, i.e., the attachment of NZVI particles on the surface of BC or in the interior pores of BC particles is still not be observed directly. If the location of NZVI is not on surface or in interior pores of BC particles, the attachment of NZVI on/or in BC cannot be employed to interpret the better reduction performance of NZVI/BC than bare NZVI for contaminants. Therefore, it’s needed to explore the location of NZVI in NZVI/BC for better understanding the improved reduction performance of NZVI/BC than bare NZVI.

Cross-sectional TEM (C-TEM) technique, first reported in 1974^32^, is conducted through directly observing the inner ultra-thin slices (with thickness less than 100 nm) of samples using P-TEM technique. This technique can eliminate the problem of superimposition of P-TEM technique by avoiding the interfering of the sample surface signals, and thus, obtain the pure signals of interior structure of the sample^33–35^. Therefore, C-TEM technique has been applied widely in characterizing of solid materials such as bulk heterojunction films^36,37^, biochar^38^, and activated carbons^39^ in previous studies. In this study, therefore, C-TEM technique was firstly employed to directly identify the interior structures of NZVI/BC, especially the micro locations of NZVI in NZVI/BC particles. The comparison of the pH-dependent percent removal curves of metal cations under the presence of solid materials with the pH-dependent precipitation curves of metal cations without solid materials, a successful method established to identify whether the removal of metal cations with solid materials is adsorption or not in our previous studies^40,41^, was employed here to explore whether the removal of Fe^2+^ or Fe^3+^ ions with BC is adsorption or not. If the two curves overlapped, the removal of Fe^2+^ or Fe^3+^ in the presence of BC should be attributed primarily to precipitation but not adsorption^40^. If the removal of Fe^2+^ or Fe^3+^ with BC is by adsorption, the pH-dependent percent removal curve should be over the precipitation curve at some given pH^41^. Reduction experiments of Fe(OH)_2_ and Fe(OH)_3_ by NaBH_4_ was also conducted here to explore whether the adsorbed Fe(OH)_2_ or Fe(OH)_3_ on BC can be reduced by NaBH_4_. These experiments and the results could be helpful for exploring the underlying mechanisms to explain the better reduction performance of NZVI/BC than bare NZVI for contaminants.

## Results and discussion

**Figure 1:**
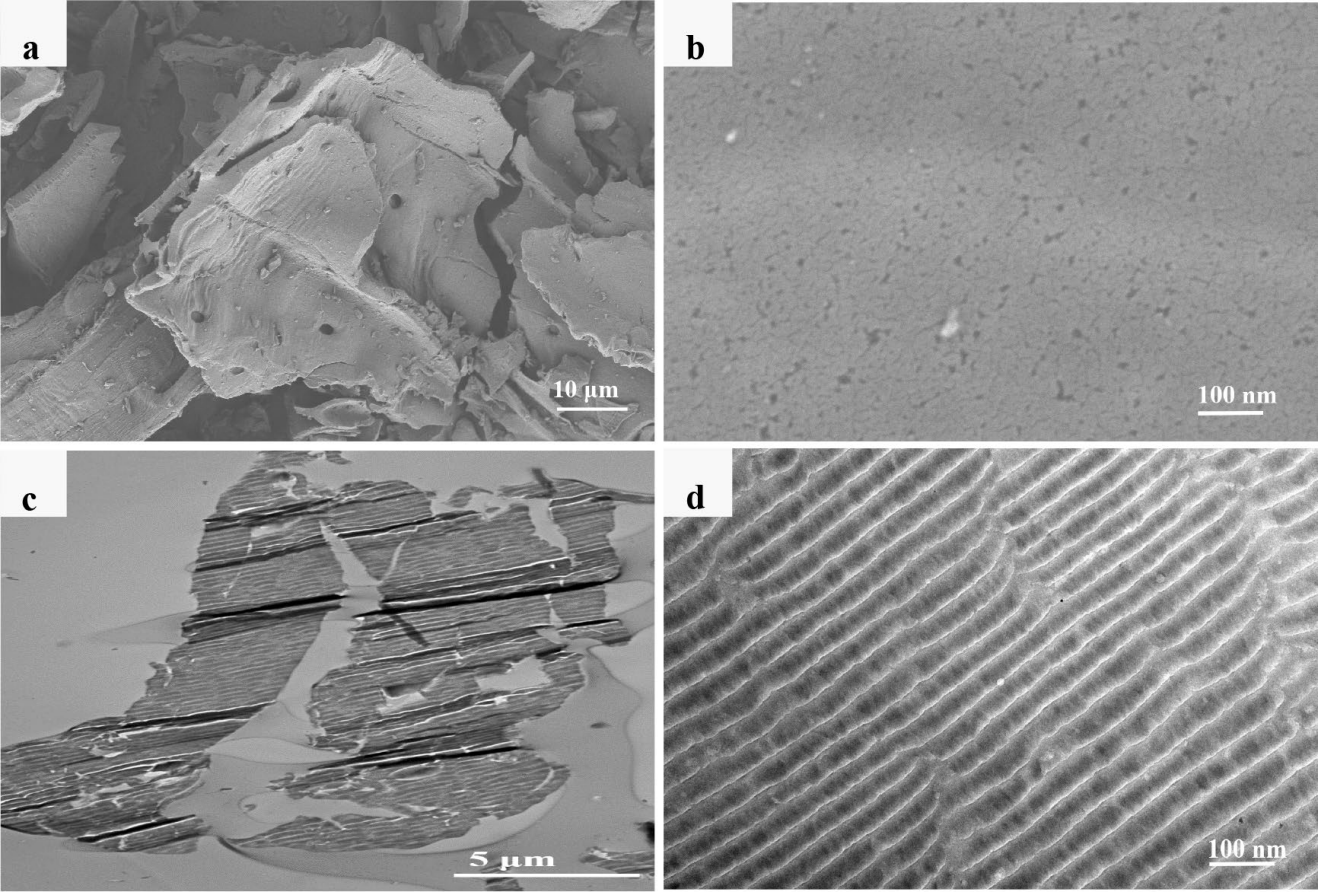
SEM images (**a**,**b**) and C-TEM images (**c**,**d**) of BC.

**Figure 2:**
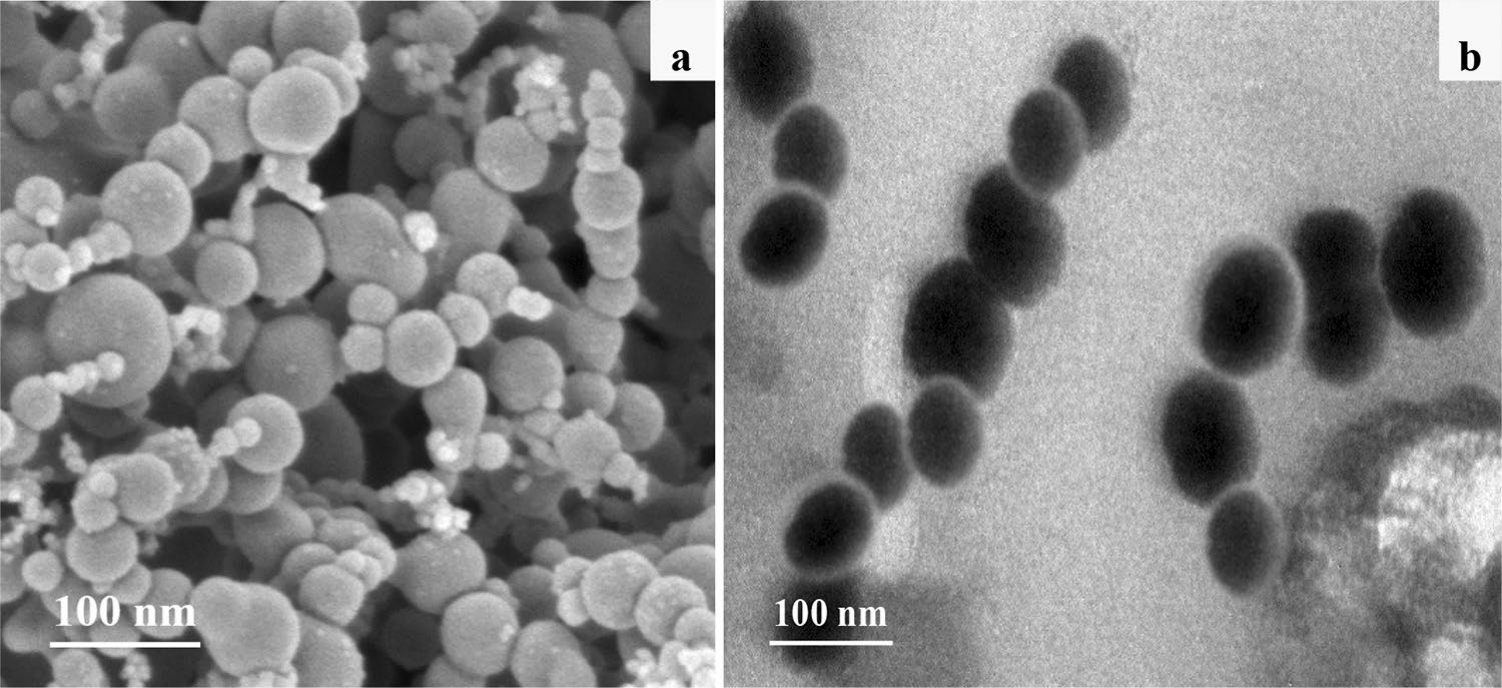
SEM image (**a**) and C-TEM image (**b**) of NZVI.

**Figure3:**
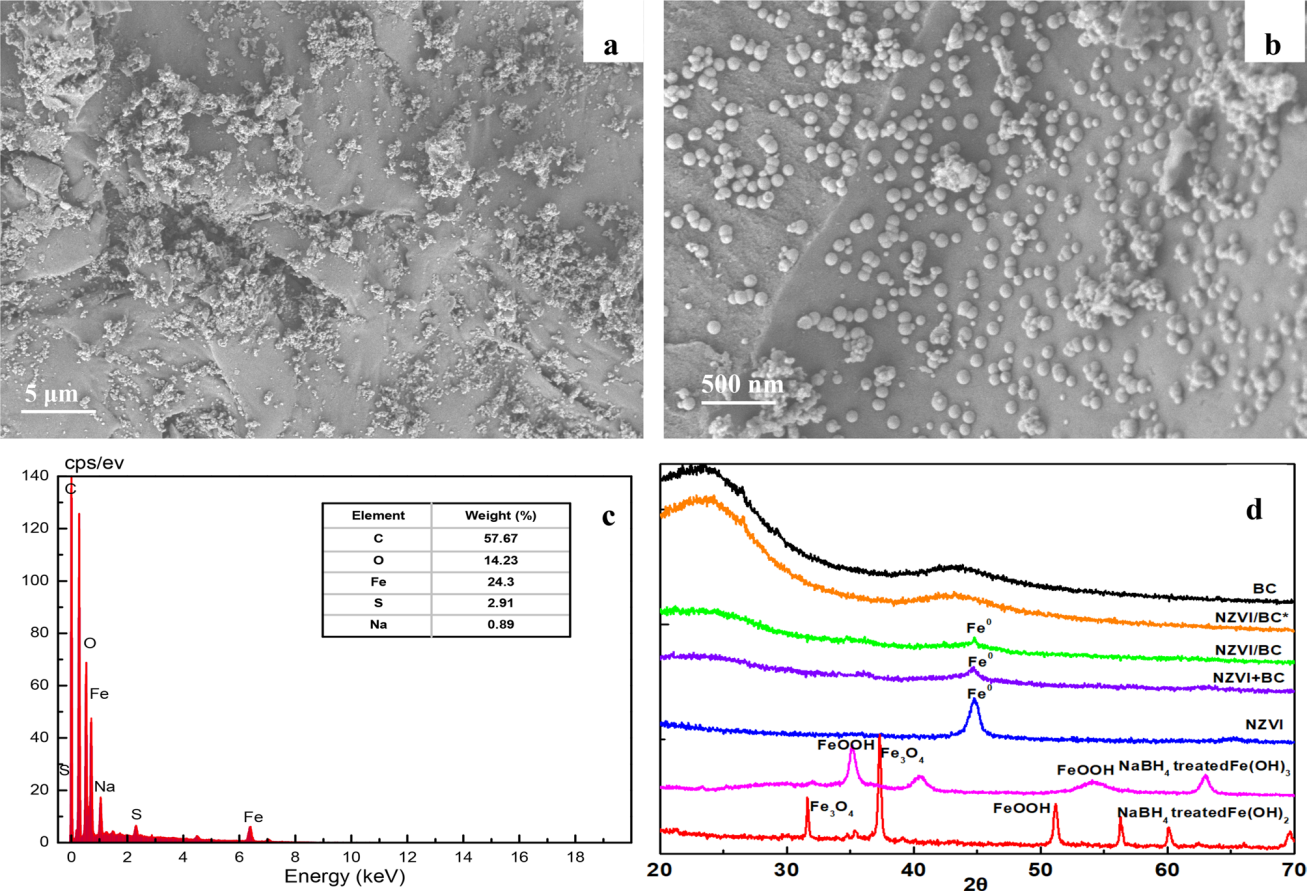
SEM images (**a**,**b**) and EDX (**c**) spectrum of NZVI/BC, as well as XRD patterns (**d**) of BC, NZVI/BC, NZVI/BC*, NZVI + BC, NZVI, NaBH_4_ treated Fe(OH)_2_ and NaBH_4_ treated Fe(OH)_3_.

**Figure 4:**
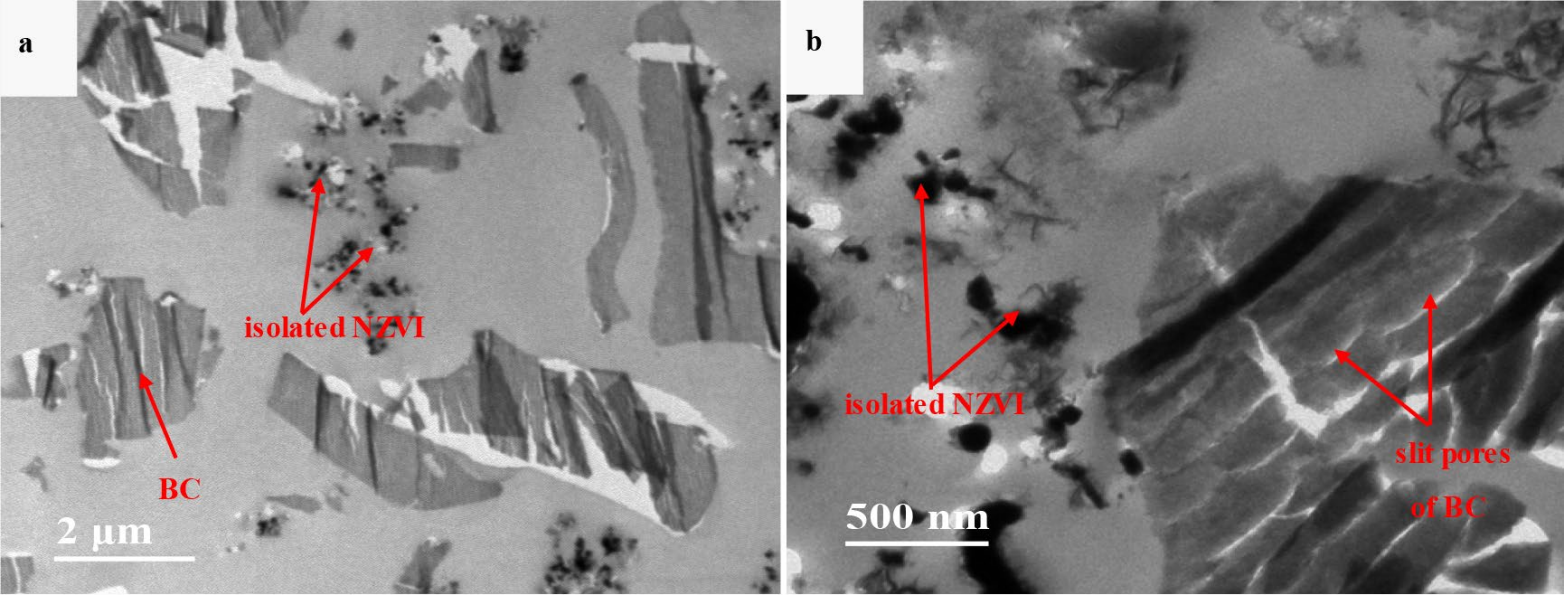
C-TEM images (**a**,**b**) of NZVI/BC.

**Figure 5:**
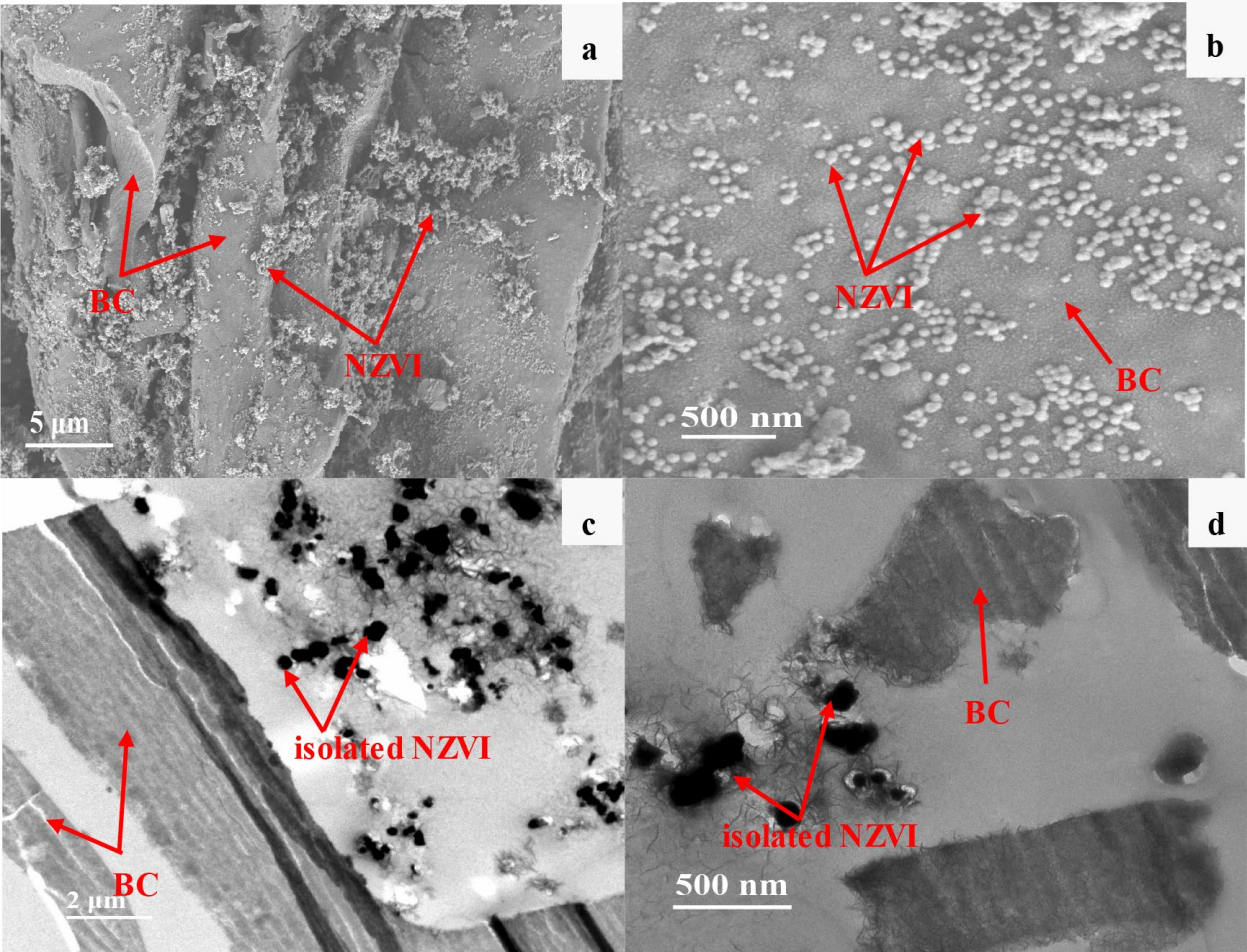
SEM (**a**,**b**) and C-TEM images (**c**,**d**) of NZVI + BC.

The surface morphology of BC particles observed by SEM technique is relatively smooth in lamellar structure (Fig. 1a), with abundant irregular pores (Fig. 1b). However, in C-TEM images (Fig. 1c,d), the slices of BC particles are in the sheet-like shape, showing abundant slit pores in uniform pore size. Bare NZVI particles in SEM (Fig. 2a) and C-TEM (Fig. 2b) images are roughly spherical and aggregated significantly to a chain-like structure, which should be attributed to the intrinsic magnetic attraction between the NZVI particles^4,5,12,42,43^. There are a lot of chain-like clusters and roughly spherical particles dispersed on BC surface in SEM images of NZVI/BC (Fig. 3a,b). These chain-like clusters and roughly spherical particles on the surface of NZVI/BC are NZVI particles, as was also observed by the SEM images of bare NZVI particles (Fig. 2a) and NZVI/BC in previous studies^11,16,18,42^. EDX spectrum of NZVI/BC shows that the main elemental composition of NZVI/BC is C, Fe and O (Fig. 3c). The homogeneous distribution of C and Fe was also verified in SEM elemental mapping of NZVI/BC (Figure S1). NZVI particles formed in NZVI/BC are identified by the characteristic peak at 44.7° (Fig. 3d) of XRD pattern of NZVI/BC. NZVI particles formed and dispersed on the surface of NZVI/BC were also observed in P-TEM images of NZVI/BC in previous studies^19,22^. However, in C-TEM images of NZVI/BC (Fig. 4a and Figure S1), NZVI particles are existed isolated from BC particles rather than attached on the surface of BC particles. The distinguished observations in C-TEM images of NZVI/BC from that in SEM and P-TEM images of NZVI/BC, could be attributed to the top surface view of SEM technique^27,29^ and the superimposition problem of P-TEM technique ^30,31^. Thus, both of SEM and P-TEM techniques cannot explore the interior structure signals of NZVI/BC particles. Moreover, NZVI particles are not observed in interior slit pores of BC particles (Fig. 4b and Figure S1), i.e., NZVI particles cannot be embedded into the interior pores of BC particles. As reported in the previous literatures^11,44,45^, the average particle size of NZVI on NZVI/BC is about 30–100 nm. In this study, the particle size of NZVI in NZVI/BC is in the range of 40–180 nm (Fig. 3b). However, BC is basically composed of micropores and mesopores with average diameter less than 10 nm^13,38,46^. The N_2_ adsorption–desorption isotherm and pore size distribution of BC in this study are shown in Figure S3. The specific surface and average pore size of BC are 579.15 m^2^/g and 2.486 nm, respectively. Therefore, NZVI particles cannot enter into interior pores of BC particles, showing the clean and slit pores without any iron particles in C-TEM images of NZVI/BC (Fig. 4). In addition, the surface morphology of NZVI/BC in SEM images (Fig. 3a,b) and inner cross section structure of NZVI/BC in C-TEM images (Fig. 4) as well as the XRD pattern (Fig. 3d) of NZVI/BC are similar to that of NZVI + BC (Figs. 3d and 5), the sample mixed from NZVI and BC directly. Therefore, NZVI/BC is similar to NZVI + BC, without attachment of NZVI on BC surface or into BC interior pores, i.e., the better reduction ability of NZVI/BC for contaminants than NZVI cannot be interpreted by the attachment of NZVI on BC surface or into BC interior pores.

**Figure 6:**
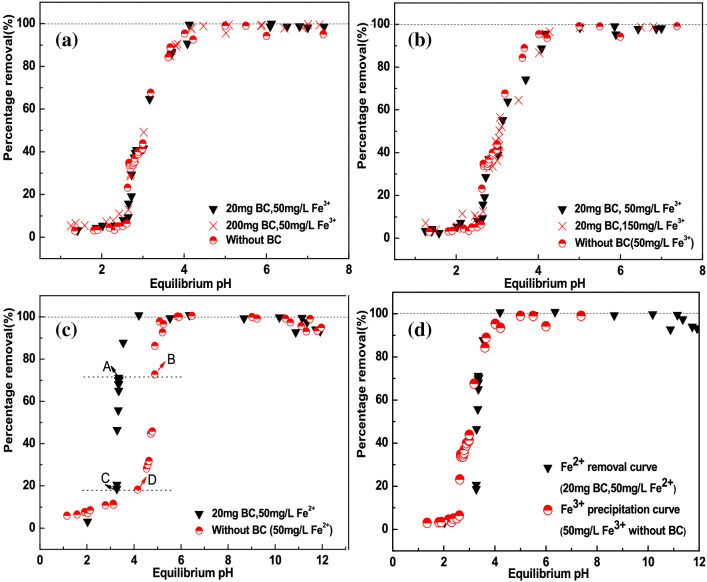
pH-dependent percent removal curves of Fe^3+^ or Fe^2+^ with or without BC. The used BC dose for Fe^3+^ are 0, 20, 200 mg in 20 mL solution (**a**), while that for Fe^2+^ are 0 and 20 mg (**c**). The employed concentration of Fe^3+^ are 50 and 150 mg/L (**b**), while that of Fe^2+^ is 50 mg/L (**c**,**d**).

Adsorption of Fe^2+^ or Fe^3+^ ions on BC and then reduction of adsorbed Fe^2+^or Fe^3+^ ions by NaBH_4_ was proposed as a possible way to load NZVI on BC surface or in BC interior pores^15,20–22^. However, we observed that the removal curves of Fe^3+^ in the presence of BC under various pH overlapped with the precipitation curve of Fe^3+^ without BC (Fig. 6a,b). Moreover, removal curves of Fe^3+^ were independent of the dosage of BC (20 and 200 mg) in Fig. 6a or initial concentration of Fe^3+^ (50 and 150 mg/L) in Fig. 6b. Therefore, the removal of Fe^3+^ ions in the presence of BC should be attributed primarily to precipitation by formation of Fe(OH)_3_ rather than adsorption on BC^40^. Although the removal curve of Fe^2+^ didn’t overlapped with the precipitation curve of Fe^2+^ without BC (Fig. 6c), it overlapped with the precipitation curve of Fe^3+^ without BC (Fig. 6d), implying that the oxidation of Fe^2+^ to Fe^3+^ and then the precipitation of Fe^3+^ may be responsible for the removal of Fe^2+^ with BC. In order to check this hypothesis, four different points (A, B, C, D) were chosen to determine the mass of Fe^2+^ and Fe^3+^ in supernatants and solids, respectively (Fig. 6c). Species distribution of Fe^2+^ and Fe^3+^ in supernatant and solids are listed in Table S1. Oxidation of Fe^2+^ to Fe^3+^ with or without BC were observed (Table S1). In the presence of BC and higher pH, more Fe^2+^ were oxidized to Fe^3+^ (Table S1). Therefore, the oxidation of Fe^2+^ to Fe^3+^ occurred in batch experiment for Fe^2+^ by BC. Characteristic peak of NZVI was not observed in XRD diffraction pattern of NZVI/BC*, a sample prepared by removing excessive free Fe^2+^ in Fe^2+^/BC system before adding NaBH_4_ (Fig. 3d), indicating almost no Fe^2+^ or Fe^3+^ adsorbed on BC. Moreover, the C-TEM diagrams of NZVI/BC* were similar to that of BC (Figures S4), indicating that no black NZVI particles observed in NZVI/BC, i.e., no Fe^2+^ or Fe^3+^ adsorption on BC observed. Therefore, Fe^2+^ and Fe^3+^ cannot be fixed on BC surface or in interior pores through adsorption to form NZVI on BC by reduction using NaBH_4_.

**Figure 7:**
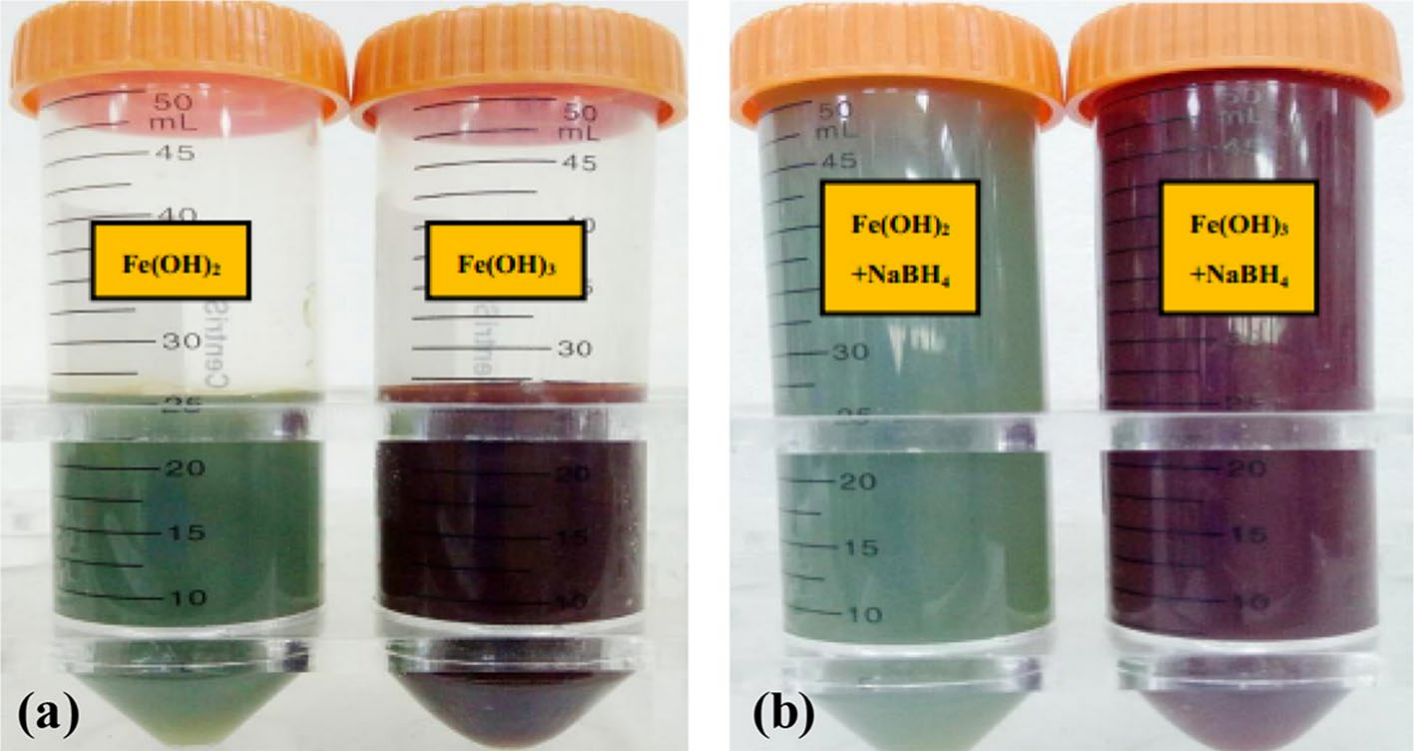
Pictures of suspensions of Fe(OH)_2_ and Fe(OH)_3_ precipitates before (**a**) and after treated with NaBH_4_ solution (**b**).

Forming of Fe(OH)_2_ or Fe(OH)_3_ precipitates, deposited on BC and then reduced to NZVI by NaBH_4_ was proposed as another possible way to load NZVI on BC surface or in BC interior pores^21,23^. However, depositing of Fe(OH)_2_ or Fe(OH)_3_ precipitates on the surface or in the interior pores of BC particles were not observed in C-TEM images of NZVI/BC* (Figure S4), showing that the surface and pores of NZVI/BC* are clean and similar to that of BC (Figure S4). Moreover, NZVI can be obtained by reducing iron salt with NaBH_4_ rather than hydroxide^4,47^, i.e., Fe(OH)_2_ or Fe(OH)_3_ cannot be reduced to NZVI by NaBH_4_. For example, the suspensions of dark green Fe(OH)_2_ precipitates and reddish brown Fe(OH)_3_ precipitates (Fig. 7a)_,_ prepared from Fe^2+^ and Fe^3+^ respectively via chemical precipitation, are not changed in color after dropping NaBH_4_ (Fig. 7b) under nitrogen atmosphere, i.e., No black ZVI particles formed after reduction treatment of Fe(OH)_2_ and Fe(OH)_3_ precipitates with NaBH_4_. The characteristic peak of NZVI was not observed in XRD diffraction patterns of the dried solid samples collected from NaBH_4_ treated Fe(OH)_2_ and NaBH_4_ treated Fe(OH)_3_ (Fig. 3d), indicating NZVI cannot be obtained from Fe(OH)_2_ and Fe(OH)_3_ precipitates by reduction using NaBH_4_. Therefore, NZVI cannot be loaded on BC via depositing of Fe(OH)_2_/Fe(OH)_3_ precipitates on surface or into interior pores of BC particles as well as reduction of Fe(OH)_2_/Fe(OH)_3_ precipitates to NZVI by NaBH_4._

Except for loading of NZVI on the surface or into the interior pores of BC, smaller size of NZVI in NZVI/BC was proposed to interpret the better reduction performance of contaminants by NZVI/BC than bare NZVI^5,22,42^. Smaller-sized NZVI is more reactive, which can be attributed to its higher surface area-to-volume ration and stronger quantum effects^4,6,11^. In this study, bare NZVI particles are aggregated into micron-size particles (Fig. 2), which is consistent with the previous reports^6,42,48^. However, the particle size of NZVI in NZVI/BC is in the range of 40–180 nm (Figs. 3b and 4), much smaller than that of bare NZVI (Fig. 2), implying that the agglomeration of NZVI particles is effectively alleviated by BC^10,22^. The alleviation of NZVI agglomeration was also observed in sample NZVI + BC, showing that the particle size of NZVI in NZVI + BC is in the range of 50–220 nm (Fig. 5). Therefore, the smaller size of NZVI in NZVI/BC than that of bare NZVI could be possibly responsible for the better reduction performance of contaminants by NZVI/BC than bare NZVI^10–12^. In previous studies^10,13,42^, the smaller size of NZVI in NZVI/BC was attributed to the attachment of NZVI on BC surface or into the interior pores of BC particles that inhibit the aggregation of NZVI. However, in this study, the observed smaller size of NZVI in NZVI/BC should be attributed to the mixing of NZVI with BC that inhibit the aggregation of NZVI, but not the attachment of NZVI on/in BC particles.

## Conclusion and outlook

Employing C-TEM technique for liquid reduction method prepared NZVI/BC, it was observed in this study that NZVI particles are existed isolated from BC particles in NZVI/BC, rather than attached on surface or in interior pores of BC particles that was observed in previous study by SEM and P-TEM techniques. This observation was also supported by the negligible adsorption of Fe^2+^/Fe^3+^ and the negligible attachment of Fe(OH)_2_/Fe(OH)_3_ precipitates on BC surface or interior pore structure of BC particles, as well as the negligible reduction of Fe(OH)_2_/Fe(OH)_3_ to NZVI by NaBH_4_. Therefore, both of the widely used SEM and P-TEM techniques have difficulty in observing the inner structure of NZVI/BC. In addition to SEM and P-TEM, C-TEM is a useful technique to characterize the interior morphology of NZVI/BC and other porous materials supported NZVI for better exploring their underlying improved reduction performance of contaminants. Besides, C-TEM can also be employed to characterize the interior morphology and configuration of other composite materials. The better reduction performance of NZVI/BC than bare NZVI could be attributed to the smaller size of NZVI particles in NZVI/BC, due to mixing NZVI with BC particles, which inhibit the aggregation of NZVI, but could not be explained by the attachment of NZVI particles on surface or into interior pores of BC particles in NZVI/BC.

## Materials and methods

### Chemicals

Ferrous sulfate heptahydrate (FeSO_4_·7H_2_O, 97%), sodium borohydride (NaBH_4_, 98%), and hydroxylamine hydrochloride (NH_2_OH·HCl, 99%) were supplied by Sinopharm Group Chemical Reagent, China. Ferric chloride hexahydrate (FeCl_3_·6H_2_O, 99%) was obtained from Aladdin Reagent Corporation.

### Preparation of BC and NZVI materials

BC was prepared from wood chips (Ningbo, Zhejiang, China) by oxygen-limited pyrolysis method^49^. Briefly, dried wood chips, grounded to pass through a 0.15 mm sieve, were packed in crucibles, covered with lids, and then, placed in a muffle furnace, pyrolyzed at 700 °C for 6 h. The prepared BC was washed with 1 mol/L HCl (m/v = 0.1 g/mL) by stirring for 24 h to remove impurities and then rinsed using deionized (DI) water to neutral pH. The obtained BC sample was dried overnight at 80 °C in an oven for usage.

Bare NZVI was synthesized using the liquid-phase reduction method^11,12,50^. Briefly, under nitrogen atmosphere, 100 mL NaBH_4_ solution (1.2 mol/L) was dropped (1–2 drops/s) into 100 mL FeSO_4_ solution (0.44 mol/L) with stirring. The black NZVI particles were separated by centrifugation for 5 min at 3000 rpm. Then, NZVI particles were washed with deoxygenated DI water, followed by ethanol, and finally dried in a vacuum oven at 60 °C for 12 h.

NZVI/BC was prepared according to the procedures reported in the previous literature^51^. Under nitrogen atmosphere, 100 mL of 0.44 mol/L FeSO_4_ solution was mixed with 5.0 g BC for 3 h. Then, 100 mL of 1.2 mol/L NaBH_4_ was added dropwise into the mixture. After reaction, NZVI/BC was collected by centrifugation at 3000 rpm for 5 min, washed with deoxygenated DI water and ethanol, and dried in a vacuum oven (60 °C, 12 h).

NZVI/BC* was prepared as follows. 100 mL of 0.44 mol/L FeSO_4_ solution was mixed with 5.0 g BC under nitrogen atmosphere for 24 h. Then the solid was collected by centrifugation at 3000 rpm for 5 min and followed by washing with deoxygenated DI water for three times to remove excess free Fe^2+^ that not adsorbed on BC. Under nitrogen atmosphere, 100 mL of 1.2 mol/L NaBH_4_ was added dropwise into the suspension of BC which was pretreated with FeSO_4_ solutions. Then, by centrifugation at 3000 rpm for 5 min, washed with deoxygenated DI water and ethanol, and dried in a vacuum oven at 60 °C for 12 h, nZVI/BC* was collected.

NZVI + BC was prepared directly by mixing BC and NZVI particles. Briefly, 1.0 g BC and 0.5 g NZVI were mixed in 30 mL ethanol in a beaker and dispersed by ultrasonic cell breaker (SCIENTZ-II D) for 10 min (20 °C, 570 W). Then, the mixed NZVI + BC sample was separated by centrifugation at 3000 rpm for 5 min and dried in a vacuum oven (60 °C, 12 h).

### Characterization of BC and NZVI materials

**Figure 8:**
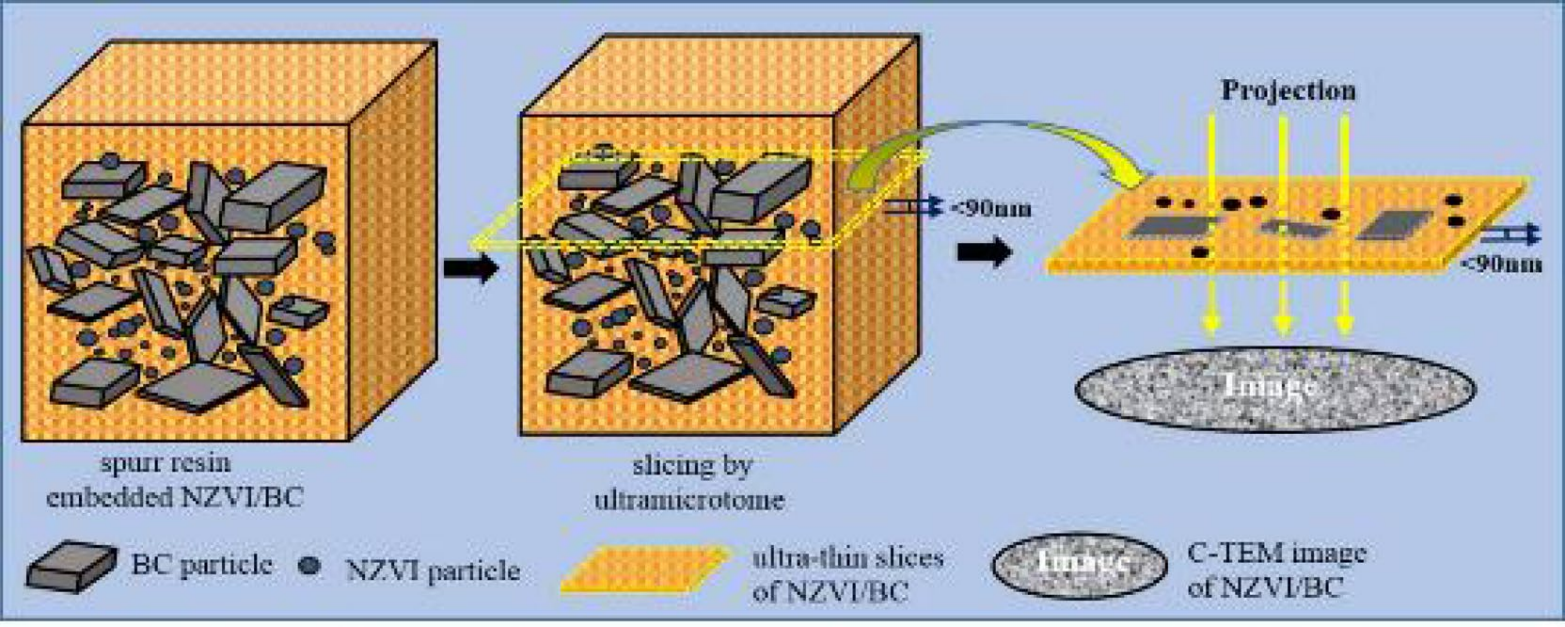
Schematic of ultra-thin slice of NZVI/BC preparation.

The inner structure information of BC and NZVI materials was observed by C-TEM technique directly^38,39^. These samples were firstly embedded into embedding agent, i.e., spurr resin, which is a mixture of 10 g vinyl cyclohexene dioxide (ERL 4221), 8 g diglycidyl ether of polypropylene glycol (DER 736), 25 g nonenyl succinic anhydride (NSA) and 0.3 g 2-dimethylaminoethanol (DMAE), purchased from SPI-Chem Supplies. Then, spurr resin embedded samples were heated at 70 °C for 12 h, and cut into ultra-thin slices with thickness less than 90 nm by ultramicrotome (Leica, EMUC7, Germany) equipped with diamond knife (Fig. 8). The slices were finally collected on copper mesh grids for observation of TEM at 200 kV (JEM1200, JEOL, Japan).

X-ray diffraction patterns of samples were obtained by an X-ray diffractometer (XRD, Philips, Netherlands) equipped with a CuKα radiation source and scanned at a speed of 2° per min. Surface morphologies of samples were examined by using a field emission SEM (FE-SEM) (SIRON, FEI, Netherlands) equipped with an energy dispersive spectroscopy (Oxford Inca EDS) at a voltage of 25.0 kV.

### Batch experiments for Fe^2+^ and Fe^3+^ by BC

Fe^2+^ and Fe^3+^ solution was prepared from FeSO_4_·7H_2_O and FeCl_3_·6H_2_O respectively. Batch experiment of Fe^2+^ and Fe^3+^ was conducted in 22 mL screw cap vials with a total solution volume of 20 mL sealed with Teflon-coated septa under ambient temperature (25 °C). Briefly, 20 mg or 200 mg BC was added into vials having 18 mL background solution with various pH value, prepared from DI water with 0.01 mol/L HCl and NaOH. Then, 2 mL Fe^2+^ or Fe^3+^ solution (500 mg/L or 1500 mg/L), was added into the vials to give the initial Fe^2+^ or Fe^3+^ concentration of 50 mg/L or 150 mg/L, respectively. The vials were capped and transferred to a horizontal shaker (DHZ-D) at 150 rpm for 48 h. After centrifugation at 3000 rpm for 5 min, supernatant was taken from vials, and filtered with a 0.22 μm needle filter to remove the solid. The pH value of supernatant was measured using a glass pH electrode (Mettler Toledo). The concentration of Fe^2+^ iron in supernatant was determined by UV-spectrophotometer (UV-2450, Shimadzu, Japan) with 1,10-phenanthroline spectrophotometric method at a maximum wavelength of 510 nm^52^. For measurement of Fe^3+^, Fe^3+^ in supernatant was reduced to Fe^2+^ firstly by NH_2_OH·HCl^53^, and then, determined by UV-spectrophotometer with 1,10-phenanthroline spectrophotometric method^52^.

The removal efficiency of Fe^2+^ or Fe^3+^ was calculated by Eq. (1):

1 $${\text{R}}\% = (C_{0} - C{\text{e}})/C_{0} *{1}00\%$$

where *C*_0_ (mg/L) is the initial concentration of Fe^2+^ or Fe^3+^ in solution, *C*e (mg/L) is the equilibrium concentration of Fe^2+^ or Fe^3+^ in solution.

### Precipitation experiments of Fe^2+^ and Fe^3+^ in aqueous phase

Precipitation of Fe^2+^ and Fe^3+^ was also conducted in 22 mL screw cap vials. 18 mL background water at various pH, prepared from DI water with 0.01 mol/L HCl and NaOH, and 2 mL Fe^2+^or Fe^3+^ solution (500 mg/L) were mixed in the vials, i.e., the initial Fe^2+^or Fe^3+^ concentration in vials was 50 mg/L. The vials were capped and transferred to a horizontal shaker (DHZ-D) at 150 rpm for 48 h under ambient temperature (25 °C). After centrifugation at 3000 rpm for 5 min, pH value and equilibrium concentration of Fe^2+^ or Fe^3+^ in supernatant were measured with glass pH electrode (Mettler Toledo) and UV-spectrophotometer (UV-2450, Shimadzu, Japan), respectively^52,53^.

### Reduction of Fe(OH)_2_ and Fe(OH)_3_ by NaBH_4_

For reduction experiments of Fe(OH)_2_ or Fe(OH)_3_ precipitates by NaBH_4,_ 10 mL 0.25 M Fe^2+^ or Fe^3+^ solution and 10 mL 1.0 M NaOH solution were mixed and then centrifuged at 3000 rpm for 5 min to collect Fe(OH)_2_ or Fe(OH)_3_ precipitates. The collected Fe(OH)_2_ or Fe(OH)_3_ precipitate was washed with deoxidizing DI water for three times to remove excessive Fe^2+^ or Fe^3+^ ions. Before reduction by NaBH_4,_ another 20 mL deoxidizing DI water was added to redisperse Fe(OH)_2_ or Fe(OH)_3_ precipitates. Then, 20 mL 1.0 M NaBH_4_ was added dropwise under nitrogen atmosphere to reduce the precipitates. After reduction reaction, solid product was collected by centrifugation at 3000 rpm for 5 min, washed with deoxidizing DI water and ethanol, and finally dried in a vacuum oven at 60 °C for 12 h.

## Supplementary Information

Supplementary Information 1.

## References

[1] Liu SC (2020). Study on influencing factors and mechanism of removal of Cr(VI) from soil suspended liquid by bentonite-supported nanoscale zero-valent iron. Sci. Rep..

[2] Ahmad M (2020). Turning date palm waste into carbon nanodots and nano zerovalent iron composites for excellent removal of methylthioninium chloride from water. Sci. Rep..

[3] Gil-Díaz M, Álvarez MA, Alonso J, Lobo MC (2020). Effectiveness of nanoscale zero-valent iron for the immobilization of Cu and/or Ni in water and soil samples. Sci. Rep..

[4] Wang CB, Zhang WX (1997). Synthesizing nanoscale iron particles for rapid and complete dechlorination of TCE and PCBs. Environ. Sci. Technol..

[5] Zhao X, Liu W, Cai Z, Han B, Qian T, Zhao D (2016). An overview of preparation and applications of stabilized zero-valent iron nanoparticles for soil and groundwater remediation. Water Res..

[6] Zou Y (2016). Environmental remediation and application of nanoscale zero-valent iron and its composites for the removal of heavy metal ions: A review. Environ. Sci. Technol..

[7] Mackenzie K, Bleyl S, Kopinke FD, Doose H, Bruns J (2016). Carbo-Iron as improvement of the nanoiron technology: From laboratory design to the field test. Sci. Total. Environ..

[8] Diao ZH (2018). Insights into the simultaneous removal of Cr^6+^ and Pb^2+^ by a novel sewage sludge-derived biochar immobilized nanoscale zero valent iron: Coexistence effect and mechanism. Sci. Total. Environ..

[9] Qian LB (2020). Field demonstration of enhanced removal of chlorinated solvents in groundwater using biochar-supported nanoscale zero-valent iron. Sci. Total. Environ..

[10] Dong HR (2017). Stabilization of nanoscale zero-valent iron (nZVI) with modified biochar for Cr(VI) removal from aqueous solution. J. Hazard. Mater..

[11] Liu CM, Diao ZH, Huo WY, Kong LJ, Du JJ (2018). Simultaneous removal of Cu^2+^ and bisphenol A by a novel biochar-supported zero valent iron from aqueous solution: Synthesis, reactivity and mechanism. Environ. Pollut..

[12] Xu J (2020). Iron and sulfur precursors affect crystalline structure, speciation, and reactivity of sulfidized nanoscale zerovalent iron. Environ. Sci. Technol..

[13] Dong HR (2017). Removal of trichloroethylene by biochar supported nanoscale zero-valent iron in aqueous solution. Sep. Purif. Technol..

[14] Liati A (2014). Electron microscopic study of soot particulate matter emissions from aircraft turbine engines. Environ. Sci. Technol..

[15] Zhu S (2017). Magnetic nanoscale zerovalent iron assisted biochar: Interfacial chemical behaviors and heavy metals remediation performance. ACS Sustain. Chem. Eng..

[16] Wei AL, Ma J, Chen JJ, Zhang Y, Song JX, Yu XY (2018). Enhanced nitrate removal and high selectivity towards dinitrogen for groundwater remediation using biochar-supported nano zero-valent iron. Chem. Eng. J..

[17] Liu WJ, Jiang H, Yu HQ (2015). Development of biochar-based functional materials: Toward a sustainable platform carbon material. Chem. Rev..

[18] Ahmed MB (2017). Nano-Fe-0 immobilized onto functionalized biochar gaining excellent stability during sorption and reduction of chloramphenicol via transforming to reusable magnetic composite. Chem. Eng. J..

[19] Shang JG, Zong MZ, Yu Y, Kong XR, Du Q, Liao QJH (2017). Removal of chromium (VI) from water using nanoscale zerovalent iron particles supported on herb-residue biochar. J. Environ. Manag..

[20] Qian LB (2017). Nanoscale zero-valent iron supported by biochars produced at different temperatures: Synthesis mechanism and effect on Cr(VI) removal. Environ. Pollut..

[21] Wang SS (2019). Biochar-supported nZVI (nZVI/BC) for contaminant removal from soil and water: A critical review. J. Hazard. Mater..

[22] Zhang DJ (2019). Enhanced nitrobenzene reduction by modified biochar supported sulfidated nano zerovalent iron: Comparison of surface modification methods. Sci. Total. Environ..

[23] Chen W, Pan L, Chen L, Wang Q, Yan C (2014). Dechlorination of hexachlorobenzene by nano zero-valent iron/activated carbon composite: Iron loading, kinetics and pathway. RSC Adv..

[24] Maillard M, Giorgio S, Pileni MP (2002). Silver nanodisks. Adv. Mater..

[25] Cao GH, Simon P, Krämer U, Wimbush SC, Holzapfel B (2004). Transmission electron microscopy and high-resolution electron microscopy study of YNi2B2C thin film on Y_2_O_3_-Buffered MgO. Chem. Mater..

[26] Wang YN, Wei J, She Q, Pacheco F, Tang CY (2012). Microscopic characterization of FO/PRO membranes-a comparative study of CLSM, TEM and SEM. Environ. Sci. Technol..

[27] Ren CL (2010). Synthesis of Ag/ZnO nanorods array with enhanced photocatalytic performance. J. Hazard. Mater..

[28] Lehman JH, Terrones M, Mansfield E, Hurst KE, Meunier V (2011). Evaluating the characteristics of multiwall carbon nanotubes. Carbon.

[29] Haigh SJ (2012). Cross-sectional imaging of individual layers and buried interfaces of graphene-based heterostructures and superlattices. Nat. Mater..

[30] Bravman JC, Sinclair R (1984). The preparation of cross-section specimens for transmission electron microscopy. J. Electron. Microsc..

[31] Liu Y, Rafailovich MH, Sokolov J, Schwarz SA, Bahal S (1996). Effects of surface tension on the dislocation structures of diblock copolymers. Macromolecules.

[32] Abrahams MS, Buiocchi C (1974). Cross-sectional specimens for transmission electron-microscopy. J. Appl. Phys..

[33] Kim DH, Jang Y, Park YD, Cho K (2006). Layered molecular ordering of self-organized poly(3-hexylthiophene) thin films on hydrophobized surfaces. Macromolecules.

[34] Akamatsu K, Shimada M, Tsuruoka T, Nawafune H, Fujii S, Nakamura Y (2010). Synthesis of pH-responsive nanocomposite microgels with size-controlled gold nanoparticles from ion-doped, lightly cross-linked poly(vinylpyridine). Langmuir.

[35] Miyachi M, Yamanoi Y, Tomo T, Nishihara H (2016). Cross-sectional TEM analysis of an ITO surface coated with photosystem I and molecular wires. J. Inorg. Organomet. P..

[36] Moon JS, Lee JK, Cho S, Byun J, Heeger AJ (2009). “Columnlike” structure of the cross-sectional morphology of bulk heterojunction materials. Nano Lett..

[37] Oleksak RP (2014). Chemical and structural investigation of high-resolution patterning with HafSOx. ACS Appl. Mater. Inter..

[38] Yang K, Jiang Y, Yang JJ, Lin DH (2018). Correlations and adsorption mechanisms of aromatic compounds on biochars produced from various biomass at 700 °C. Environ. Pollut..

[39] Yang K, Zhu LH, Yang JJ, Lin DH (2017). Adsorption and correlations of selected aromatic compounds on a KOH-activated carbon with large surface area. Sci. Total. Environ..

[40] Yang K, Wei W, Qi L, Wu WH, Jing QF, Lin DH (2014). Are engineered nanomaterials superior adsorbents for removal and pre-concentration of heavy metal cations from water?. Rsc Adv..

[41] Yang K (2015). Sorption of Cu^2+^ on humic acids sequentially extracted from a sediment. Chemosphere.

[42] Wang SS (2017). The sorptive and reductive capacities of biochar supported nanoscaled zero-valent iron (nZVI) in relation to its crystallite size. Chemosphere.

[43] Cao Z, Li H, Xu X, Xu J (2020). Correlating surface chemistry and hydrophobicity of sulfidized nanoscale zerovalent iron with its reactivity and selectivity for denitration and dechlorination. Chem. Eng. J..

[44] Zhu HJ, Jia YF, Wu X, Wang H (2009). Removal of arsenic from water by supported nano zero-valent iron on activated carbon. J. Hazard. Mater..

[45] Lv X, Xu J, Jiang G, Xu X (2011). Removal of chromium(VI) from wastewater by nanoscale zero-valent iron particles supported on multiwalled carbon nanotubes. Chemosphere.

[46] Devi P, Saroha AK (2014). Synthesis of the magnetic biochar composites for use as an adsorbent for the removal of pentachlorophenol from the effluent. Bioresour. Technol..

[47] Brown HC, Brown CA (1962). A simple preparation of highly active platinum metal catalysts for catalytic hydrogenation. J. Am. Chem. Soc..

[48] Li J, Fana MJ, Li M, Liu X (2020). Cr(VI) removal from groundwater using double surfactant-modified nanoscale zero-valent iron (nZVI): Effects of materials in different status. Sci. Total. Environ..

[49] Yang K, Yang JJ, Jiang Y, Wu WH, Lin DH (2016). Correlations and adsorption mechanisms of aromatic compounds on a high heat temperature treated bamboo biochar. Environ. Pollut..

[50] Stefaniuk M, Oleszczuk P, Ok YS (2016). Review on nano zerovalent iron (nZVI): From synthesis to environmental applications. Chem. Eng. J..

[51] Hussain I (2017). Insights into the mechanism of persulfate activation with nZVI/BC nanocomposite for the degradation of nonylphenol. Chem. Eng. J..

[52] Wu J, Zheng H, Zhang F, Zeng RJ, Xing BS (2019). Iron-carbon composite from carbonization of iron-crosslinked sodium alginate for Cr(VI) removal. Chem. Eng. J..

[53] Wylie EM, Olive DT, Powell BA (2016). Effects of titanium doping in titanomagnetite on neptunium sorption and speciation. Environ. Sci. Technol..

