# Supplementary material for: Re-recognizing micro locations of nanoscale zero-valent iron in biochar using C-TEM technique
Source: Sci Rep. 2021 Mar 3;11:5037. doi: 10.1038/s41598-021-84685-w (PMC7930034; doi:10.1038/s41598-021-84685-w)
Supplement: Supplementary file 1 — Supplementary Information 1. [file 41598_2021_84685_MOESM1_ESM.pdf]

Supporting Information

Re-recognizing Micro Locations of Nanoscale Zero-valent Iron in Biochar Using C-TEM technique

Kun Yang[1,2,3*], Jialu Xu[1,2,3], Ming Zhang[4] & Daohui Lin[1,2,3]

[1] Department of Environmental Science, Zhejiang University, Hangzhou 310058, China

[2] Key Laboratory of Environmental Pollution and Ecological Health of Ministry of Education, Hangzhou 310058, China

[3] Zhejiang Provincial Key Laboratory of Organic Pollution Process and Control, Hangzhou 310058, China

[4] Department of Environmental Engineering, China Jiliang University, Hangzhou, 310028, China

[*]Corresponding author (Kun Yang). Tel: 86-571-88982589; Fax: 86-571-88982590; E-mail: kyang@zju.edu.cn

Supporting Information consists of 7 pages, including1 text, 1 table, and 4 figures.

Table of contents

Text 1. Analysis methods of Fe^{2+}/Fe^{3+} in supernatants and solids in the precipitation experiments.

Table S1. Species distribution of Fe^{2+}/Fe^{3+} in supernatants and solids in the precipitation experiments.

Figure S1. SEM image (a) and SEM elemental mapping (b) of NZVI/BC.

Figure S2. C-TEM images of NZVI/BC prepared at different times (Figure S2a, b, c) and with various magnification (e.g., Figure S2a, d, g)

Figure S3. N_2 adsorption–desorption isotherm (a) and pore size distribution (b) of BC.

Figure S4. C-TEM images of NZVI/BC* (a, c, e) and BC (b, d, f).

Text 1. Analysis methods of Fe^{2+}/Fe^{3+} in supernatants and solids in the precipitation experiments.

The mass of Fe^{2+} in supernatants was determined by UV-spectrophotometer with 1,10-phenanthroline spectrophotometric method at a maximum wavelength of 510 nm. Sum mass of Fe^{2+} and Fe^{3+} in supernatants was determined by UV-spectrophotometer following hydroxylamine hydrochloride reduction. The mass of Fe^{3+} in supernatants was obtained by subtracting the mass of Fe^{2+} in supernatants from sum mass of Fe^{2+} and Fe^{3+} in supernatants. Precipitated Fe^{2+} and Fe^{3+} in solids were digested with 6M HCl firstly and then measured by UV-spectrophotometer as well as the determination process of Fe^{2+} and Fe^{3+} in supernatants.

	Fe^{2+} in supernatant (mg)	Fe^{3+} in supernatant (mg)	Fe^{2+} in solid (mg)	Fe^{3+} in solid (mg)	Oxidation degree $Fe^{3+}/(Fe^{2+}+Fe^{3+})$ 100%
A	0.026	0.256	0.092	0.578	87.6
B	0.214	0.076	0.052	0.512	68.9
C	0.444	0.38	0.002	0.156	54.6
D	0.562	0.146	0.182	0.0774	19.4

Table S1. Species distribution of Fe^{2+}/Fe^{3+} in supernatants and solids*

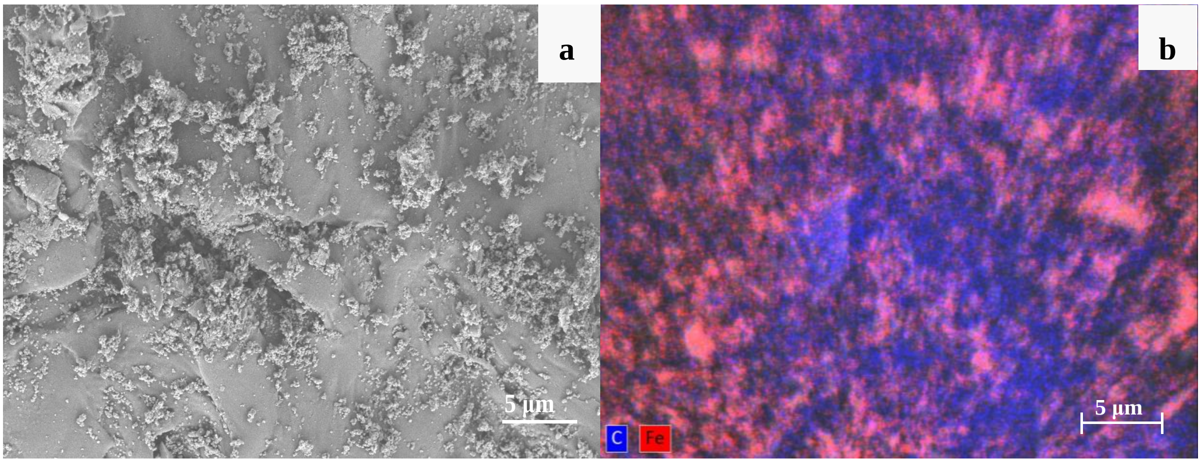

Figure S1. SEM image (a) and SEM elemental mapping (b) of NZVI/BC.

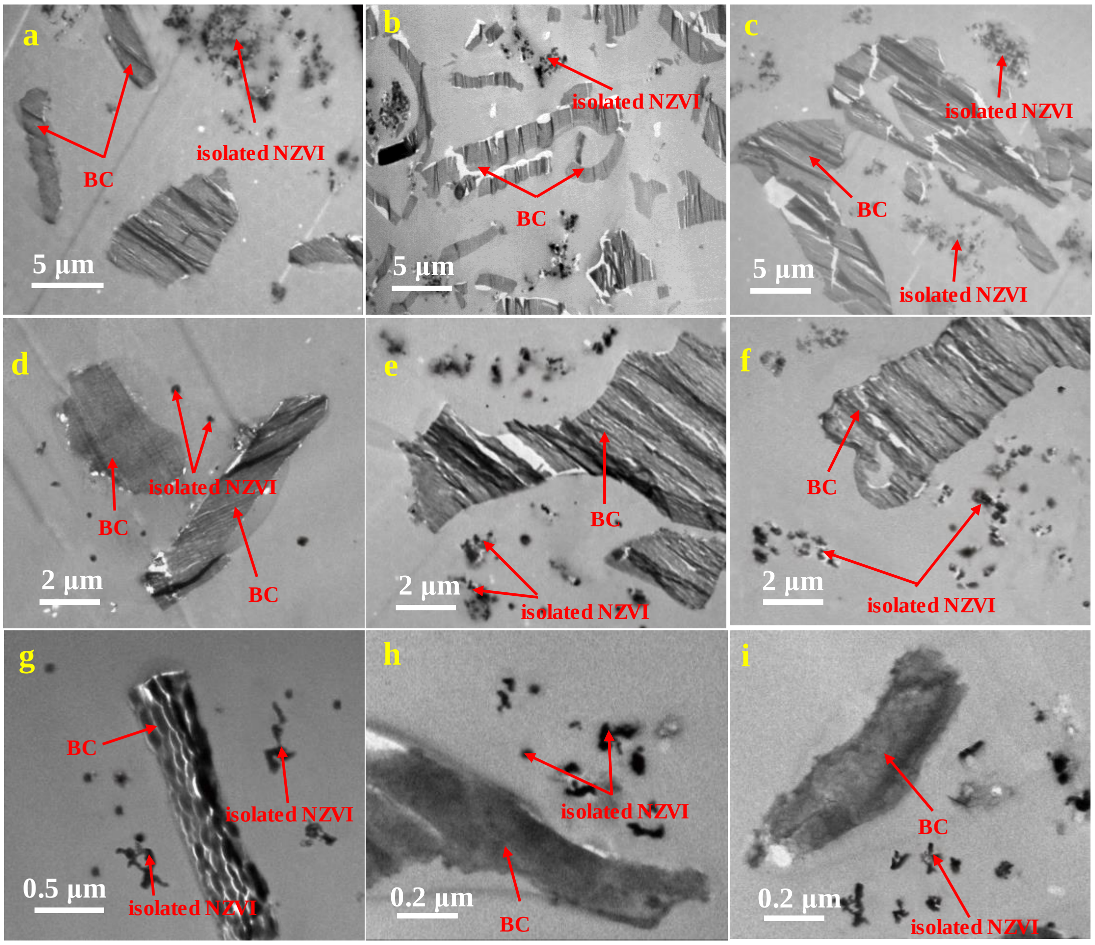

Figure S2. C-TEM images of NZVI/BC prepared at different times (Figure S2a, b, c) and with various magnification (e.g., Figure S2a, d, g)

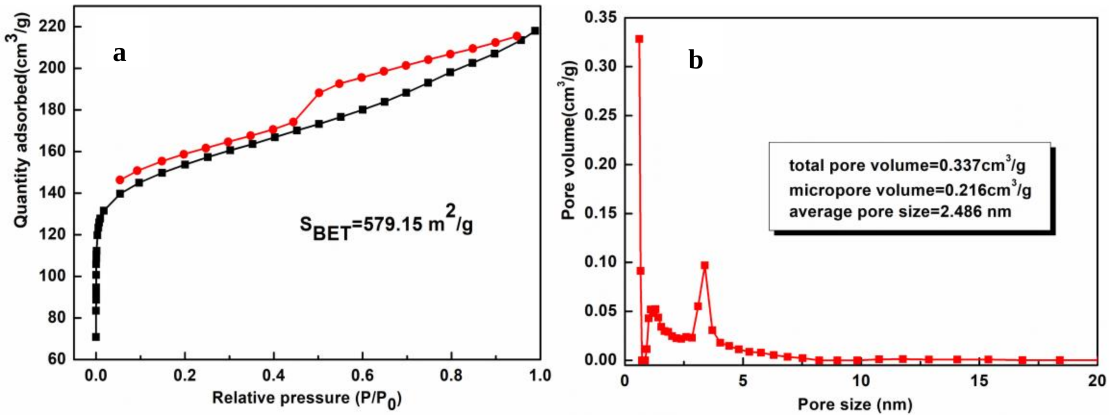

Figure S3. N_2 adsorption–desorption isotherm (a) and pore size distribution (b) of BC.

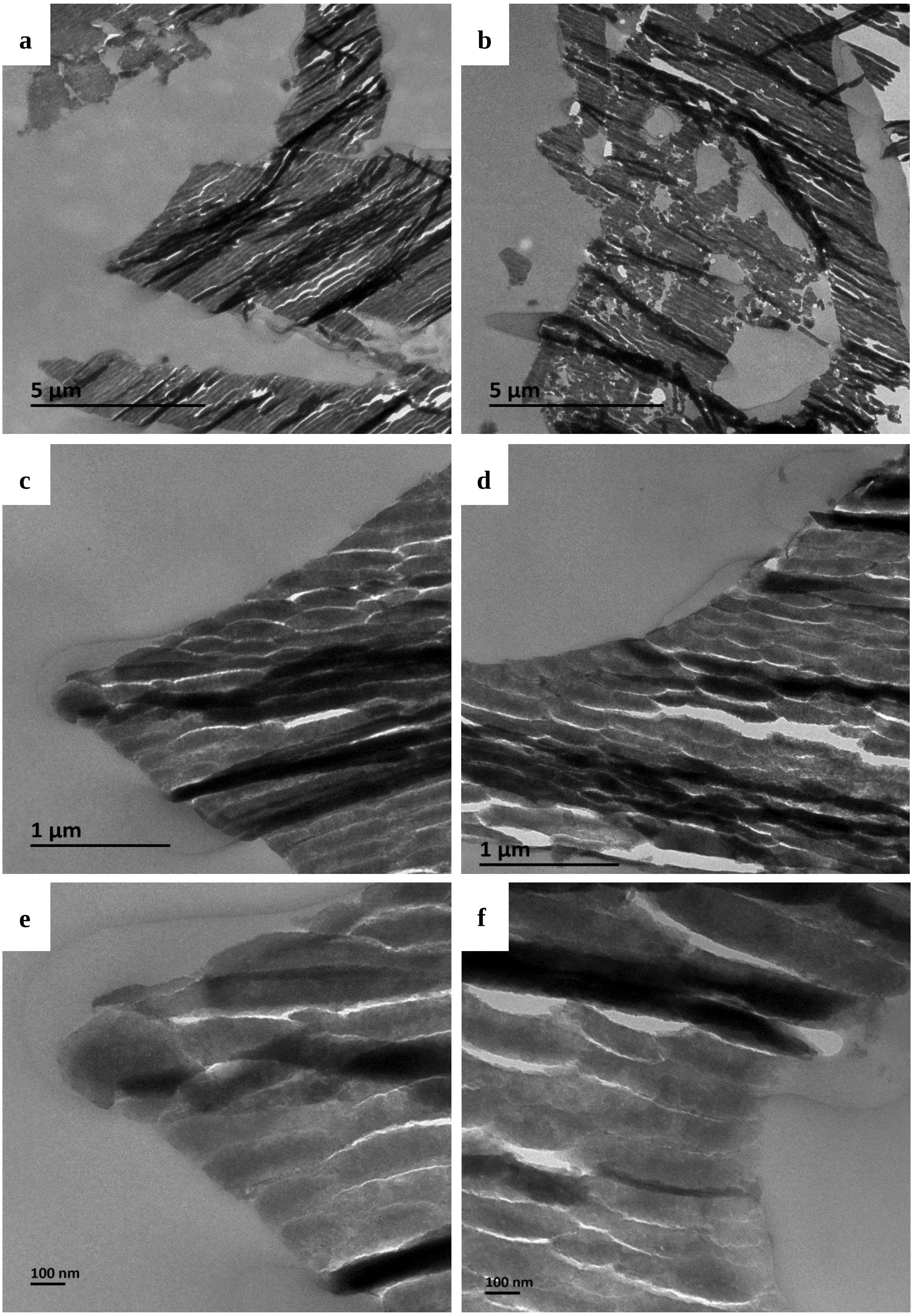

Figure S4. C-TEM images of NZVI/BC* (a, c, e) and BC (b, d, f).
